# Developing equity-focused interventions for maternal and child health in Nigeria: an evidence synthesis for policy, based on equitable impact sensitive tool (EQUIST)

**DOI:** 10.11604/pamj.2019.34.158.16622

**Published:** 2019-11-25

**Authors:** Chigozie Jesse Uneke, Issiaka Sombie, Henry Chukwuemeka Uro-Chukwu, Ermel Johnson

**Affiliations:** 1 African Institute for Health Policy and Health Systems, Ebonyi State University, PMB 053 Abakaliki, Nigeria; 2 West African Health Organization, 175, Avenue Ouezzin Coulibaly, 01 BP 153 Bobo Dioulasso 01, Burkina Faso

**Keywords:** EQUIST, maternal, child, health, Nigeria

## Abstract

**Introduction:**

Among the most critical health systems components that requires strengthening to improve maternal, newborn and child health (MNCH) outcomes in Nigeria is the concept of equity. UNICEF has designed the equitable impact sensitive tool (EQUIST) to enable policymakers improve equity in MNCH and reduce disparities between the most marginalized mothers and young children and the better-off.

**Methods:**

Using the latest available DHS data sets, we conducted EQUIST situation and scenario analysis of MNCH outcomes in Nigeria by sub-national categorization, wealth and by residence. We then identified the intervention package, the bottlenecks and strategies to address them and the number of deaths avertible.

**Results:**

EQUIST profile analysis showed that the number of under-five deaths was considerably higher among the poorest and rural population in Nigeria, and was highest in North-West region. Neonatal causes, malaria, pneumonia and diarrhoea were responsible for most of the under-five deaths. Highest maternal mortality was recorded in the North-West Nigeria. Ante-partum, intrapartum and postpartum haemorrhages and hypertensive disorder, were responsible for highest maternal deaths. EQUIST scenario analysis showed that an intervention package of insecticide treated net can avert more than 20,000 under-five deaths and delivery by skilled professionals can avert nearly 17,000 under-five deaths. While as many as 3,370 maternal deaths can be averted by deployment of skilled professionals.

**Conclusion:**

Scaling up integrated packages of essential interventions across the continuum of care, addressing the human resource shortages in rural area and economic/social empowerment of women are policy recommendations that can improve MNCH outcomes in Nigeria.

## Introduction

In Nigeria maternal and child health outcomes remain unacceptably poor, largely due to the weak health systems. Consequently, maternal and child health status in the country remains one of the worst in Africa and has not improved substantially, and in some areas of the country, has worsened over the past decade [1]. There exists a wide variation in maternal mortality ratio (MMR) across the six Nigeria geo-political zones, with the northern zones generally having worst maternal, newborn and child health (MNCH) indicators than the southern zones [1]. With the population of up to 186 million, Nigeria has about 2.5% of the population of the world and 10% of all maternal and under-five deaths, translating into more than 50 000 maternal and more than 1 million newborn, infant, and child deaths annually [2-4]. Nigeria loses 2,300 under-five year olds and 145 women of childbearing age every day, making the country the second largest contributor to the under-five and maternal mortality in the world [1]. Many of these deaths which occur during pregnancy, labor and delivery are preventable, but the coverage and quality of health care services in Nigeria continue to fail women and children [1].

Kana and co-workers, who reviewed maternal and child health interventions in Nigeria from 1990-2014, noted that since documentation of national MNCH statistics began in the early 1990s in Nigeria, poor MNCH indicators have been a recurring public health challenge [5]. Available reports have shown that more than a quarter million neonates die in Nigeria each year, representing about 700 neonates per day [6]. In Nigeria, a number of factors have been strongly linked with high mortality of neonates including mother's age, maternal illness, lack of antenatal care, low birth weight, birth asphyxia and prematurity [7,8]. Annually, up to 529,000 maternal deaths are recorded globally, and of this number, about 52,900 Nigerian women are estimated to die from complications associated with pregnancy, making the risk of a Nigerian woman dying from pregnancy and child birth to be 1 in 13 [9]. HIV/AIDS (5%), Malaria (11%), obstructed labour (11%), unsafe abortion (11%), toxaemia/eclampsia/hypertension anaemia (11%), infection (17%), and haemorrhage (23%), are responsible for most of the maternal deaths in Nigeria [2-4]. Also contributing to the high maternal mortality in Nigeria are socio-economic factors including: lack of awareness about complications in pregnancy; need to seek medical intervention early; lack of transportation to the health facilities where maternal healthcare can be provided; inability to pay for services, etc. [2,4,9,10]. The use of insecticide treated bed-nets increased from 8% in 2008 to 50% in 2013, but malaria contributes some 30% to childhood mortality [4]. AIDS, lower respiratory tract infections and diarrheal diseases are among the leading causes of years of life lost [9]. Since 2009 the Boko Haram group has led an insurgency that has seen thousands killed and led to severe humanitarian and health crises in particular in the North-East and North-West regions of Nigeria.

Among the most critical health systems components that requires strengthening to improve MNCH outcomes in Nigeria is the concept of equity. The importance of equity consideration in evidence-informed policymaking and interventions to improve MNCH in Nigeria cannot be overstated. Findings from a number of studies from low and middle-income countries (LMICs) have consistently shown that interventions leading to decrease in maternal and child mortality are accompanied by increased inequity in health outcomes between the rich and the poor [11-14]. Based on these findings, the United Nations Children's Fund (UNICEF) is strongly promoting an “equity-focused” approach in which health interventions are targeted at the poorest and the underserved population, rather than the “mainstream approach” where scaling-up of health interventions favours the wealthier population [12]. UNICEF is also currently advocating for equitable investment in health interventions in LMICs targeting MNCH since practical, high-impact and low-cost health interventions, extended to the most deprived and marginalized populations have the potential to avert more maternal and child mortality more cost-effectively [14].

As part of her effort to support the global campaign on equity focused investment in health interventions, the UNICEF developed an online equitable impact sensitive tool (EQUIST) to enable the MNCH policymakers and other stakeholders improve equity and address health disparities between the most marginalized and wealthier populations [15,16]. EQUIST helps policy makers select strategies that balance the principles of equity, effectiveness and efficiency by leading them through a logical process to identify the most rational and cost-effective solutions for their context [17]. The purpose of this evidence synthesis for policy is to use EQUIST to provide reliable evidence, on equity-focused interventions and recommendations that will inform policy development to improve MNCH outcomes in Nigeria.

## Methods

### Setting

Nigeria has a total area of 923,768 km^2^ and is located on the Gulf of Guinea of West Africa and lies between latitudes 4° and 14°N, and longitudes 2° and 15°E. The country is divided into six geopolitical zones including North-West, North-Central, North-East, South-West, South-East and South-South and comprises 36 states and the Federal Capital Territory, Abuja as the capital [18]. The states are divided into 774 local government areas (LGAs). With approximately 186 million inhabitants in 2016, Nigeria is the most populous country in Africa and the seventh most populous country in the world [19]. Being the most populous country with high fertility rate, Nigeria has third-largest young population in world, after India and China, with up to 44% of the population under 15 years of age [20]. Nigeria is the largest economy in Africa, with a GDP greater than USD 500 billion and steadily grew to over 7 percent per annum between 2005 and 2014, but this growth has been slower in 2015 [21]. Ironically, poverty is still pervasive in Nigeria, where recent figures indicate 68% of the population lives on less than US$1.25 a day [20].

The Nigeria health profile is shown in Table 1. Great disparities in health status exist, across the states and geopolitical zones of the country and disease aetiology is linked to social determinants such as socio-economic status, education, gender inequality, as well as poor access to water, sanitation and hygiene [20]. Health care delivery in Nigeria is a concurrent responsibility of the three tiers of government in the country (federal, state and LGAs), as well as the private sector. Nigeria health systems was ranked 187^th^ in the world in 2000 [22] but within the last 15 years, various health indicators have shown steady, albeit slow, improvement.

**Table 1:** Health profile of Nigeria

Country parameters	Nigeria
World bank income group	Lower-middle-income
Total population in thousands	185,990 (2016)
% Population under 15 (2015)	44
% Population over 60 (2015)	4.5
Life expectance at birth (2015)	54.5 (Both sexes), 53.4 (Male), 55.6 (Female)
Neonatal mortality per 1000 live births (2015)	34.3 [25.3-46.6]
Under-five mortality rate per 1000 live births (2015)	108.8 [83.4-139.7]
Infant mortality rate per 1000 live births (2015)	69.0 [54.8-86.2]
Maternal mortality ratio per 100 000 live births (2015)	814 (596-1180)
Lifetime risk of maternal death (1 in N) (2010)	29
Total fertility rate (per woman) (2011)	5.5
Stillbirth rate (per 1000 total births) (2009)	42
Adolescent birth rate (per 1000 women) (2006)	123
% DTP3 Immunization coverage among 1-year-olds (2014)	66
% Births attended by skilled health workers (2013)	35.2
Infants exclusively breastfed for first 6 months of life (%) (2013)	17
Density of physicians per 1000 population (2009)	0.408
Density of nurses and midwives per 1000 population (2008)	1.605
Total expenditure on health as % GDP (2014)	3.7
General govt. expenditure on health as % of total government expenditure (2014)	8.2
Private expenditure on health as % of total expenditure on health (2014)	74.9
Adult (15+) literacy rate total (2007-2012)	61
Population using improved drinking-water sources (%) (2015)	68.5 (Total), 57.3 (Rural), 80.8 (Urban)
Population using improved sanitation facilities (%) (2015)	25.4 (Total), 32.8 (Urban) 29.0 (Rural)
Poverty headcount ratio at $1.25 a day (PPP) (% of population) (2011)	54.4
Human Development Index rank (2014)	152

### EQUIST situational analysis

We used the 2013 DHS data sets for Nigeria which are the latest pre-loaded in EQUIST to perform both situational and scenario analysis. The analysis was conducted as instructed in the EQUIST user guide [23]. Using the sub-national (geo-political zones), wealth (richest to poorest quintiles) and residence (urban and rural) categorization, we performed EQUIST situational (profile and frontier) analysis to determine maternal, neonatal and under-five mortality in Nigeria. The EQUIST profile analysis is categorized into sector and theme. The sector category is further divided into demographic and epidemiological parameters, while the theme category is divided into family care practices, preventive services and curative services. We assessed the under-five mortality and the neonatal mortality in Nigeria and related them to the key drivers, the underlying factors and the scale of the inequities. This was achieved by the analysis of the demographic parameters of the sector category. The analysis was used to provide information on the following: (a) the part of Nigeria that recorded the highest child (under-five and neonatal) mortality and considered the most deprived in terms of MNCH interventions; (b) the most disadvantaged or vulnerable children; i.e. how deprivation is affected by various drivers such as wealth, geography, and location; (c) the health conditions that cause excess mortality among the most disadvantaged populations; and (d) the health interventions that are linked to this excess mortality in the most deprived areas. We analysed the epidemiological parameters of the sector category, and identified the main diseases responsible for under-five, neonatal and maternal mortality. We also analysed the theme category, to determine the level of effective package coverage of family care practices, preventive services and curative services. We related these to the various zones in Nigeria to identify the population that is mostly affected by sub-national categorization, wealth and residence.

### EQUIST scenario analysis

We performed EQUIST scenario analysis for the North-West region of Nigeria. First, we assessed the main epidemiological causes of under-five mortality and maternal mortality in the region (prematurity and asphyxia). Second, we identified interventions considered as priorities that can address the epidemiological causes of under-five and maternal mortality, grouped in “packages” under family care practices (ITN ownership and use), preventive services (DPT3 immunization), and curative services (delivery by skilled professionals). Third, we determined the major possible bottleneck (geographical accessibility) that can constitute potential impediment to the identified intervention. We assessed the severity, how they affect utilization of the intervention packages and coverage and the strategies to address them. Four, we analysed the enabling environment that can facilitate the strategies for addressing bottlenecks from the perspective of the health systems building block components (task shifting, redeployment/relocation existing staff, non-facility service provision, lay/community health worker service delivery, contracting out). Five, we performed impact analysis to determine the number of avertible under-five and maternal deaths.

## Results

### Outcome of EQUIST situational analysis

**Figure 1:**
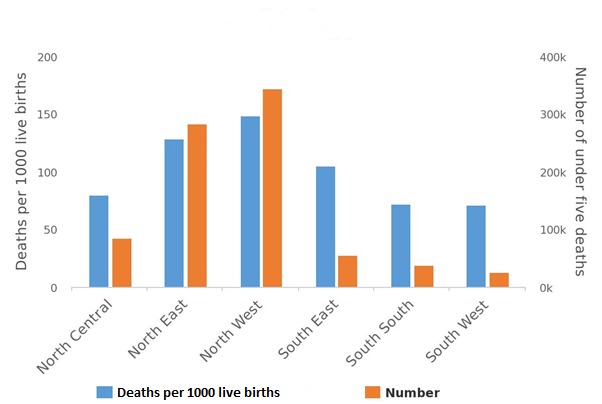
EQUIST situational analysis of Nigeria under-five mortality by province (geopolitical zones)

The result of the Nigeria EQUIST profile analysis showed that under-five mortality rate was highest in the North-West region (149/1000 live births) and the mortality figure was more than double the number obtained in the region with the lowest under-five mortality in Nigeria (South-West region) (72/1000 live births). The under-five mortality rate in the North-West region was also considerably higher than the Nigeria national average (117/1000 live births) (Figure 1). The outcome of the assessment of under-five mortality by wealth and residence also showed that mortality was considerably higher in the rural population and the poorest population.

**Figure 2:**
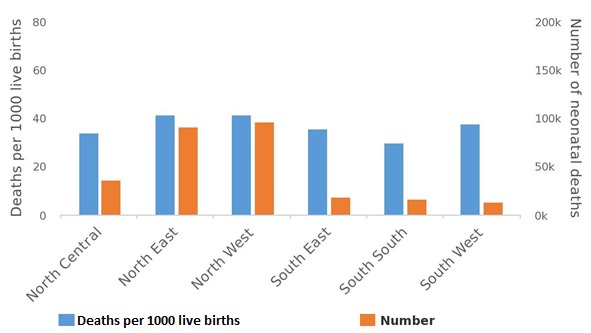
EQUIST situational analysis of Nigeria neonatal mortality by province (geopolitical zones)

**Figure 3:**
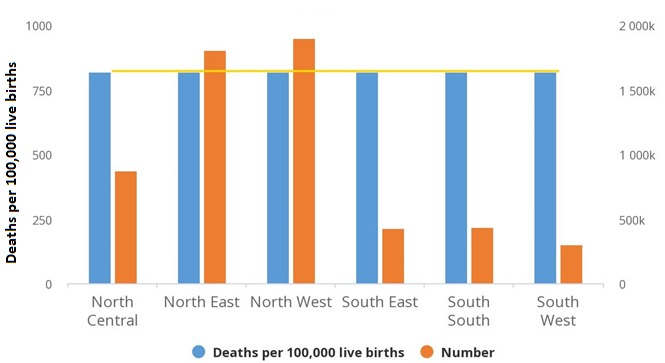
EQUIST situational analysis of Nigeria maternal mortality by province (geopolitical zones)

**Figure 4:**
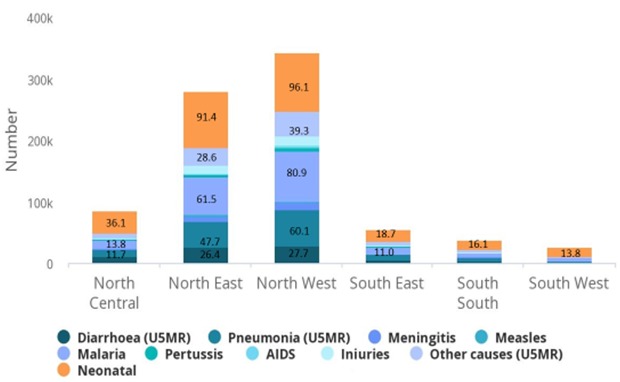
Nigeria under-five mortality by cause and by province (geopolitical zones)

**Figure 5:**
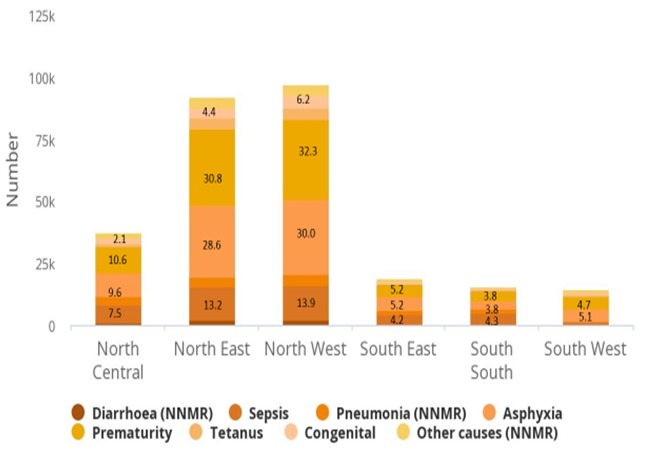
Nigeria neonatal mortality by cause and by province (geopolitical zones)

The EQUIST profile analysis showed that the South-West Nigeria recorded the least neonatal mortality number of 13,872 while the highest mortality number of 97,046 was recorded in the North-West region (Figure 2). The neonatal mortality rate among the rural population (43/1000 live births) and poorest population (44/1000 live births) was higher than the national average (36/1000 live births). The EQUIST profile analysis showed that maternal mortality ratio for Nigeria was 821/100,000 live births but the highest maternal deaths was recorded in the North-West region (1,897,017) with the least from the South-West (299,711) (Figure 3) The EQUIST profile analysis of under-five mortality by epidemiological cause is presented in Figure 4. North-West followed by the North-East of Nigeria recorded the highest mortality of the different causes of under-five deaths. Neonatal causes were responsible for most of the deaths in North-West and the North-East regions (41/1000 live births). Malaria was the second largest cause of under-five deaths in North-West (35/1000 live births) and North-East (28/1000 live births) (Figure 4). Most of the under-five deaths in Nigeria occurred in the rural compared to the urban areas and among the poorest. Prematurity and asphyxia were the leading causes of neonatal deaths in all the regions of Nigeria but the North-West and North-East recorded the highest neonatal mortality rate (Figure 5). Most of the neonatal deaths were recorded in the rural compared to the urban areas and among the poorest.

The outcome of analysis of level of effective package coverage of family care practices (ITN ownership and use), preventive services (immunization) and curative services (delivery by skilled professional) is presented in Table 2 The lowest national percentage of ITN coverage was recorded in North-East (10%) and North-West (11%) which were below the national average (13%). Also, the lowest coverage for immunization was also recorded in the North-East (21%) and North-West (14%) which were below the national average of 38%. The percentage coverage for delivery by skilled professional was lowest in both North-East (18%) and North-West (11%) with the national average at 28% and the South-West, South-East and North-Central regions recording the highest percentage of 27% (Table 2).

**Table 2:** Percentage of health intervention effective coverage by sub-national regions, residence and wealth in Nigeria (2013 DHS)

Situational description	Family Care Practices			Preventive Services	Curative Services		
	WASH (Improved water source)	ITNs (ITN ownership)	NIF (Excl breast feeding)	Immunization Plus (DTP3)	IMNCI (Oral antibiotic case mgt)	Delivery by skilled professionals (Essential care)	EMONC (Case mgt of prematurity)
National average	59	13	17	38	19	28	4
North-Central	53	13	17	44	23	27	8
North-East	49	10	17	21	16	18	1
North-West	57	11	17	14	19	11	<1
South-East	68	18	21	81	21	27	28
South-South	67	15	21	70	18	25	10
South-West	65	15	17	66	24	27	25
Rural	54	13	17	25	16	20	1
Urban	86	13	21	62	25	27	16
Poorest	36	9	17	7	14	6	<1
Richest	84	12	17	80	35	27	29

**Key:** WASH=water, sanitation and hygiene; ITN=insecticide treated bednets; Excl=exclusive; DTP3=diphtheria-tetanus-pertussis; mgt=management; EMONC=emergency obstetric and newborn care; DHS= demographic health survey

### Outcome of EQUIST scenario analysis

**Figure 6:**
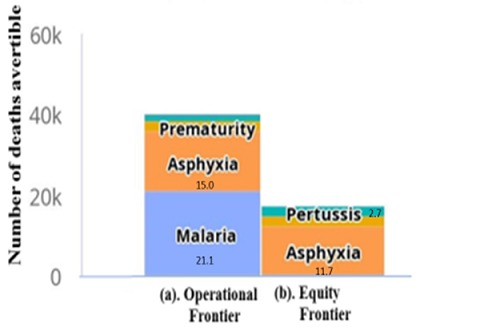
North West zone of Nigeria under-five mortality avertible by epidemiological causes by operational and equity frontier

**Figure 7:**
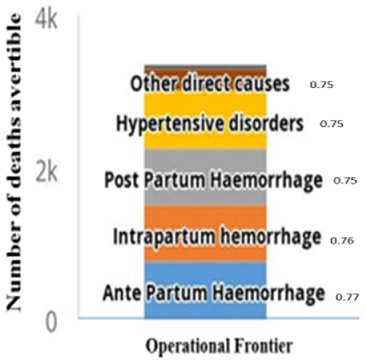
North West zone of Nigeria maternal mortality avertible by epidemiological causes by operational frontier

The number of avertible under-five and maternal deaths by operational frontier (if the deprived population coverage value was equal to the best performing countries) and equity frontier (the non-deprived population coverage value) are shown in Figure 6 and Figure 7. A total of 21,051 under-five deaths caused by malaria, and 15,002 deaths caused by asphyxia can be averted by operational frontier. An intervention package of ITN deployment can avert 22,225 under-five deaths and delivery by skilled professionals can avert 16,927 deaths (Figure 6). A total number of 765 maternal deaths caused by ante-partum haemorrhage and 757 caused by intrapartum haemorrhage can be averted by operational frontier (Figure 7). While as many as 3,370 maternal deaths can be averted by deployment of skilled professionals (Figure 7).

## Discussion

The outcome of the EQUIST situational analysis clearly showed that the North-West and North-East have the worst maternal and child health indicators in Nigeria. The North-East and North-West regions also had the lowest coverage of health interventions especially the preventive curative services. However, result showed that generally in Nigeria, the poorest and the rural dwellers recorded higher number of maternal and child deaths. These outcomes are consistent with findings from a number of previous studies in Nigeria [24-27]. According to the Nigeria National Population Commission (NPC), there is a high degree of socio-economic and cultural variations across the six geopolitical zones of Nigeria [28]. The northern region of Nigeria particularly the North-East and North-West geopolitical zones have the highest illiteracy level, polygamous marriage, early marriage (teenage pregnancy), poor utilization of modern health facility, proportion of rural residence and poverty [28]. The North-West and North-East have also been experiencing severe impact of religious insurgency by the deadly Boko Haram, which has further led to significant humanitarian and health crises in the region. These factors have been shown to be responsible for the very poor maternal and child health indicators in the North-West and North-East Nigeria [24-28]. One of the outstanding qualities of the EQUIST is its ability to estimate the number of avertible deaths if the deprived population coverage value was equal to the best performing countries and if the deprived population coverage value was equal to the non-deprived population coverage value [23,29]. In this study, we have used EQUIST to determine the number of avertible maternal and under-five deaths in the North-West Nigeria if appropriate cost-effective intervention packages are deployed through evidence-informed policy. Since this evidence synthesis is designed to provide decision makers some policy options for addressing the MNCH challenges in Nigeria, the policy options will also be of value to other African countries with similar setting as Nigeria. We have summarized six policy options based on the works of Black and colleagues [30], Santi and Weigert [31] and UNICEF EQUIST publications [14-16].

***Policy option 1:*** scaling up integrated packages of essential interventions across the continuum of care. Nigeria can actually accelerate progress in improving MNCH outcomes by scaling up integrated packages of essential interventions across the continuum of care. The intervention packages described in EQUIST capable of improving MNCH include: (i) Family Care Practices (WASH, ITN/Environmental safety, Neonatal and infant feeding and care); (ii) Preventive services (Family planning, antenatal care, immunization plus); (iii) Curative services (Integrated management of neonatal and childhood illness IMNCI, delivery by skilled professional, emergency obstetrics and neonatal care EMONC). According to Black and colleagues [30], scaling up the type of interventions described in the EQUIST, plus folic acid before pregnancy, and child health from the existing rate of coverage to 90 percent would avert 149,000 maternal deaths; 849,000 stillbirths; 1,498,000 neonatal deaths; and 1,515,000 child deaths. It is therefore imperative to determine coverage determinant, the causes of bottleneck and strategy to address them if scaling up of interventions must be achieved. EQUIST serves as a valuable tool for this purpose. It is pertinent to state that interventions and strategies for improving MNCH outcomes are closely related and must be provided through the approach of a continuum of care [32]. The importance of integrated intervention model cannot be overstated since there is a global awareness of the promotion of efficient and yet cost-effective strategies to improve maternal and child health in low income settings. There is sufficient evidence showing that when linked together and included as integrated programs, these interventions can lower costs, promote greater efficiencies, and reduce duplication of resources [33]. There is therefore need for continuous efforts to identify synergies and integrate these interventions across the continuum of care. It is also important for establishment of consensus among the key stakeholders on the content of MNCH packages of interventions at each level of the health systems so as to facilitate the scaling-up of these interventions; and identifying research gaps in the content of core packages of interventions [32]. Instead of fragmenting intervention processes and competing calls for maternal and child policy and programme, attention should shift towards an MNCH continuum of care with focus on universal coverage of effective interventions and building resilient, comprehensive and responsive health systems [34].

***Policy option 2:*** increasing budget allocation to the health sector to address the significant material and human resource shortages especially in rural and underserved area. The EQUIST analysis indicated that the rural and the poorest populations have the worst maternal and child health indices in Nigeria, implying that sufficient resources are not invested in health in the rural and underserved areas. According to Santi and Weigert [31], the poor health and medical infrastructure network in West African countries reflects the inequalities in terms of access to health, especially between the rural and urban areas and between the poorest and the richest. If this problem must be addressed, adequate funding must be allocated to the health sector to engage more health workers in order to attain the critical threshold of 23 health workers (physicians, nurses, midwives) per 10,000 inhabitants stipulated by WHO as necessary to deliver essential MNCH services [35]. It is a well-established fact that the health sector is skilled-labour-intensive and the increase in health workforce is very crucial to the overall improvement in the performance of the health systems. In this domain, emphasis must be placed on territorial equity in order to address the human resource shortage in rural areas, where the poorest people live but which still harbour the greatest health risks [31]. Underfunded investments in MNCH are part of the impediment towards the implementation of feasible and cost-effective interventions targeted at reducing maternal and child mortality [36, 37]. Interventions that have been proven to be very effective are often lacking in LMICs, there is therefore the need for increased investment in health system infrastructure, capacity enhancement of health workers, and patient enlightenment for these are critical to improving health outcomes for mothers and newborns [38]. In order to address the insufficiently diversified and autonomous financing of health, it is important to invest the growth dividend in health and explore other avenues to raise additional resources to fund the health sector [31].

***Policy option 3:*** creating enabling environment that will facilitate private sector investment in the health sector. One of the ways to address the equity issue and bridge the gap among the wealth quintiles as indicated by the EQUIST analysis, is to encourage more private sector investment in health since the government sector cannot meet all the health needs of the population. It is important to institute mechanisms that will facilitate the investment into the health sector by the organized private sector in partnership with government authorities. The government should provide enabling environment that will be attractive enough to private investors in the modern medicine sector so they can invest in it. Santi and Weigert [31], argued that the main obstacle to the involvement of private investors is the low solvency of demand, despite the growing need for increasingly diversified healthcare. Differences in essential newborn care at birth between private and public health facilities are well established [39]. In some countries including Kenya and Nigeria, available reports show that considerably more deliveries occur in private clinics and hospitals than in public ones [40-43]. Among the mandates of the newly launched Every Newborn Action Plan is coordinated support and effort amongst private sector providers of delivery services and newborn care [44]. In Nigeria, private maternity care was the preferred place of delivery because of the problems associated with public owned hospitals including low quality of facilities, absence of staff, poor perceived quality, long waiting times, and high costs [40]. It is therefore imperative for the enactment of policies that will facilitate the engagement of the private sector to increase accessibility to reproductive and child health care [39]. The Forum on Engaging the Private Sector in Child Health (FORUM-EPSCH) in an earlier report advised governments of low income settings to take urgent steps to engage the private sector in order to achieve health goals especially as they affect child health [45]. The FORUM-EPSCH [45], stressed the importance of identifying suitable catalysts for developing public-private partnerships in the health sector including enabling policy environment, coalition building among health professional associations, increased funding, and investing in monitoring and evaluation.

***Policy option 4:*** establishing effective Health insurance schemes through strong health systems reforms. Health insurance scheme is one of the intervention packages with a very high potential of improving the MNCH as shown by EQUIST. The objective of establishing functional and effective health insurance schemes is not to follow a universal coverage model that exists elsewhere, but rather to design one that is adapted to the needs of the region and evolves as progress is achieved [31]. In Africa, irrespective of the existence of multi-ethnic, cultural, tradition, lingual and religious diversity and differences, there is still a very strong social bonding which manifests in the establishment of homogenous social groups. In Nigeria as in many other African countries, any health insurance scheme that is anchored on social bonding culture of the population is most likely to succeed. This is important because available reports have indicated that the so-called formal health insurance scheme has not really worked in most of the African countries [46-48]. Of all the types of health insurance schemes, the Community-Based Health Insurance (CBHI) and Mutual Health Insurance (MHI) schemes have been shown to have the highest potential of success in a population where strong social bonding exists [49-51]. A typical example of success is the case of Senegal where the pooling of resources helped to increase the solvency of the poorest patients, especially in rural areas, where mutual health organizations (les mutuelles de santé) have fuelled attendance in health institutions and a decline in health expenditure among the poorest members of the various communities [31,52].

***Policy option 5:*** focusing the health systems on diseases and risks that affect the largest number of people and the poorest. Through EQUIST, the diseases and risks that are responsible for the largest number of maternal and child deaths were identified. It is important to concentrate on high-impact interventions that have proven successful in reducing maternal and child mortality, although such interventions have been identified, they are still under-utilized and inadequately financed [31]. Since the EQUIST analysis has clearly identified the populations with worst health indices, special effort must be made to reduce inequalities by ensuring that these most disadvantaged populations benefit from healthcare investments. It is crucial to understand the main causes of deaths to enable improved planning and targeting of interventions. EQUIST analysis indicated that the four diseases responsible for most of the under-five deaths are neonatal causes, malaria, pneumonia and diarrhoea, while the four diseases responsible for the highest maternal mortality included ante-partum haemorrhage, intrapartum haemorrhage, postpartum haemorrhage, and hypertensive disorder. Targeting interventions toward major causes of death and risk factors is a critical step toward achieving success [53]. Because much of the burden of maternal and child mortality and ill health is concentrated among the poorest populations, the highest mortality is observed among the marginalized and poor, who frequently reside in remote and rural areas with limited access to health care services [53,54]. In a recent fact sheet on reducing mortality among children [55], the WHO calls on member states to address health equity through universal health coverage so that all children irrespective of status (whether among the rural or poorest population) are able to access essential health services without undue financial hardship.

***Policy option 6:*** improving the status of women through economic empowerment and making their health well-being an utmost priority. Empowerment of women through access to health and education will not only reduce maternal and child mortality but will also facilitate the reduction of the fertility rate. Creating opportunities for women to be economically and socially empowered will enable them to lead meaningful careers and earn resources to adequately take care of their health. Accessibility to healthcare is one of the bottlenecks highlighted by the EQUIST analysis and this can be partly addressed by economically empowering women. This is because a woman who is economically empowered will have the resources that will enable her seek adequate healthcare. According to Santi and Weigert [31], the demographic dividend would have increased considerably if women had greater access to education and health and the goals to be achieved are the reduction of fertility and procreation risks, increase of the average age of marriage and the introduction of women into the labour market [31]. In an earlier report, UNICEF argued that helping governments provide a quality primary school education, a UNICEF priority, will benefit maternal and infant health particularly education for girls [56]. UNICEF also noted the following [56]: (i) educating girls for six years or more drastically and consistently improves their prenatal care, postnatal care and childbirth survival rates; (ii) educating mothers also greatly cuts the death rate of children under five; (iii) educated girls have higher self-esteem, are more likely to avoid HIV infection, violence and exploitation, and to spread good health and sanitation practices to their families and throughout their communities. In a recent report by the United Nations Foundation (UNF) [57], on private sector action for women's health and empowerment, a call was made for the recognition of the centrality of gender equality and the health and rights of girls and women as emphasized in SDG 5.

## Conclusion

As the knowledge of the importance and application of EQUIST is not yet wide-spread in Africa [29], the present study is the first attempt to use the tool to provide an evidence synthesis for policy on equity-focused approach to health interventions to improve MNCH in Nigeria. Policymakers in LMICs are continuously faced with the challenge of addressing multiple health problems in their countries with limited resource. EQUIST remains a valuable tool that can supply reliable information based on country DHS which enables decision makers to prioritize vulnerable populations, priority interventions, and gain a balanced understanding of the broad health system issues that will need to be addressed in order to reduce health disparities in their countries [23]. Capacity building of researchers and policymakers on the use of EQUIST is highly recommended. Stakeholders in MNCH in LMICs are strongly encouraged to make use of EQUIST as it has proved to be a reliable tool that will help to address the equity issues regarding maternal and child health interventions in low income settings.

### What is known about this topic

- The North-West and North-East regions of Nigeria have the worst maternal and child health indicators;
- Among the most critical health systems components that requires strengthening to improve maternal and child health outcomes is the concept of equity;
- Interventions leading to decrease in maternal and child mortality is accompanied by increased inequity in health outcomes between the rich and the poor.

### What this study adds

- EQUIST can be used to determine the number of avertible maternal and child deaths in order to guide policy development of equity focused intervention;
- EQUIST can provide valuable country-specific evidence on practical, high-impact, and, low-cost health interventions to improve maternal and child health;
- Equitable investment in health interventions extended to the most deprived populations have the potential to avert more maternal and child mortality more cost-effectively.

## Competing interests

The authors declare no competing interests.
